# LASER server: ancestry tracing with genotypes or sequence reads

**DOI:** 10.1093/bioinformatics/btx075

**Published:** 2017-02-14

**Authors:** Daniel Taliun, Sonia P Chothani, Sebastian Schönherr, Lukas Forer, Michael Boehnke, Gonçalo R Abecasis, Chaolong Wang

**Affiliations:** 1 Department of Biostatistics and Center for Statistical Genetics, University of Michigan School of Public Health, Ann Arbor, MI, USA; 2 Computational and Systems Biology, Genome Institute of Singapore, Singapore, Singapore; 3 Division of Genetic Epidemiology, Department of Medical Genetics, Molecular and Clinical Pharmacology, Medical University of Innsbruck, Innsbruck, Austria

## Abstract

**Summary:**

To enable direct comparison of ancestry background in different studies, we developed LASER to estimate individual ancestry by placing either sezquenced or genotyped samples in a common ancestry space, regardless of the sequencing strategy or genotyping array used to characterize each sample. Here we describe the LASER server to facilitate application of the method to a wide range of genetic studies. The server provides genetic ancestry estimation for different geographic regions and user-friendly interactive visualization of the results.

**Availability and Implementation:**

The LASER server is freely accessible at http://laser.sph.umich.edu/

**Supplementary information:**

Supplementary data are available at *Bioinformatics* online.

## 1 Introduction

Advancing genetic studies of rare variants will require very large sample sizes. Achieving these large sample sizes is challenging both because of the need to combine samples and data across multiple sources but also because of the need to guard against population structure, which can lead to spurious signals in genetic association tests. Typically, large studies estimate genetic ancestry of study participants and use the results to control for population structure or focus analyses on matched subsets of the data (Price *et al.*, 2010). With large amounts of genetic data from many studies, there is a pressing need for tools that can provide comparable ancestry estimates using different types of genetic data and different sets of variants. We have developed the LASER method to infer ancestry places array-genotyped or sequenced individuals in a predefined reference ancestry space (Wang *et al.*, 2014, 2015). The resulting ancestry estimates are directly comparable across studies, as long as the same reference space is used in the LASER analysis.

Here, we develop a web server that allows researchers to estimate and compare genetic ancestry of genotyped and sequenced samples from different studies without pooling raw data, facilitating ancestry matching and collaboration across studies. The ancestry information can be useful for deciding which samples to include in joint association analysis or in further sequencing or genotyping experiments.

## 2 Implementation

The server is based on the LASER method, which can estimate ancestry using either genotypes or sequence reads (Supplementary Data). A key component of LASER is the ancestry reference panel: a heavily genotyped dataset of diverse populations. LASER applies principal components analysis (PCA) on the ancestry reference panel to construct a K-dimensional ancestry space S, which defines a common ancestry coordinate system for samples from different studies. To assign coordinates to a single study individual, LASER uses variants shared between this individual and the N reference panel members to perform a PCA of the N + 1 individuals and obtains the K’-dimensional (K’≥K) PCs space S'. LASER then performs a projection Procrustes analysis (Gower and Dijksterhuis, 2004) to find a set of transformations that project the N reference individuals from S' to S. The transformations maximize the Procrustes similarity between the projected coordinates and coordinates for reference samples in S. Finally, LASER uses these transformations to place the study individual from S' into S. The accuracy of the placement is partly reflected by the Procrustes similarity *t*, a score specific to each study individual. This procedure repeats until all study individuals are mapped to the same space S, regardless of differences in data types and variant sets. Importantly, the LASER method avoids shrinkage of projected coordinates that is common in other projection PCA analyses.

The LASER server currently includes three built-in ancestry reference panels: a worldwide panel to estimate continental ancestry (the HGDP dataset, including 938 individuals from 53 populations; Li *et al.*, 2008), a European panel to estimate fine-scale ancestry within Europe (the POPRES dataset, including 1385 individuals from 37 populations; Novembre *et al.*, 2008), and an Asian panel aggregated from five studies (Li *et al.*, 2008; Teo *et al.*, 2009; The 1000 Genomes Project Consortium, 2015; Xing *et al.*, 2010, 2013) to estimate fine-scale ancestry within Asia (836 individuals from 43 populations). To improve ancestry estimation, we expanded each of these panels to millions of SNPs by imputation (Das *et al.* 2016; The 1000 Genomes Project Consortium, 2015). The ancestry reference coordinates for each panel are pre-computed using only the directly genotyped SNPs to avoid potential artifacts introduced by imputation.

Selecting an appropriate ancestry reference panel is critical for LASER. When an individual’s ancestry is not represented in the reference panel, LASER may cluster the individual with reference populations of a distant genetic background, yielding misleading results (Wang *et al.*, 2015). A good practice is to start with a worldwide reference panel and gradually focus on relevant regional panels. To address this issue, we propose a novel statistic Z to help diagnose if a reference panel is appropriate by comparing each study individual’s genetic variance with his nearest neighbors in the reference space (Supplementary Data). We showed that our proposed Z score is highly informative when a European reference panel is mistakenly used for non-European samples (Supplementary Fig. S1).

The LASER server has a user-friendly web interface based on the Cloudgene platform (Schönherr *et al.*, 2012) where users can select a relevant ancestry panel and upload their data. The server accepts standard VCF files for genotype data and a matrix format to store read counts and estimated per base error rates from BAM files for sequence data; a companion utility is available for users to generate the input files from their BAM files. To facilitate quick exploration of ancestry, the LASER server generates both tabular summaries and interactive 2D/3D visualizations of the estimated coordinates. The interactive features include zooming, rotating, panning and displaying in a dynamic pie chart the ancestry composition of the *k* nearest neighbors for any selected individual.

## 3 Example

**Fig. 1:**
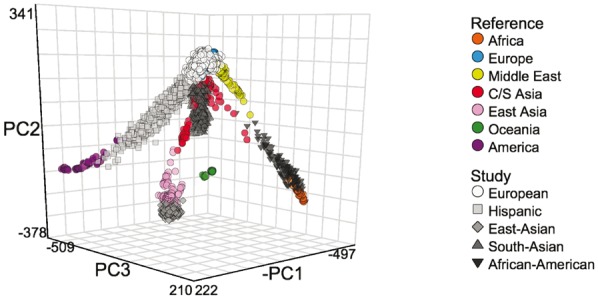
Ancestry estimation on the HGDP worldwide map for 12 940 WES samples from the T2D-GENES and GoT2D studies. This figure was exported from the 3D interactive visualization on the LASER server (http://laser.sph.umich.edu/example)

We tested the LASER server on 12 940 exomes sequenced at ∼80X depth (WES) from the T2D-GENES and GoT2D studies (Fuchsberger *et al.*, 2016). These data include five ancestry groups: European, East Asian, South Asian, Hispanic and African American. After uploading a VCF file of genotypes, the LASER server automatically identified 12 719 SNPs overlapping between the T2D-GENES/GoT2D data and the non-imputed HGDP panel, which defines a worldwide ancestry space. LASER analysis (K’=20, K = 4) suggested this was sufficient to accurately estimate continental ancestry (average *t *=* *0.998). We observed five clusters in a 3D visualization of the top PCs, corresponding to the five ancestry groups (Fig. 1).

**Fig. 2:**
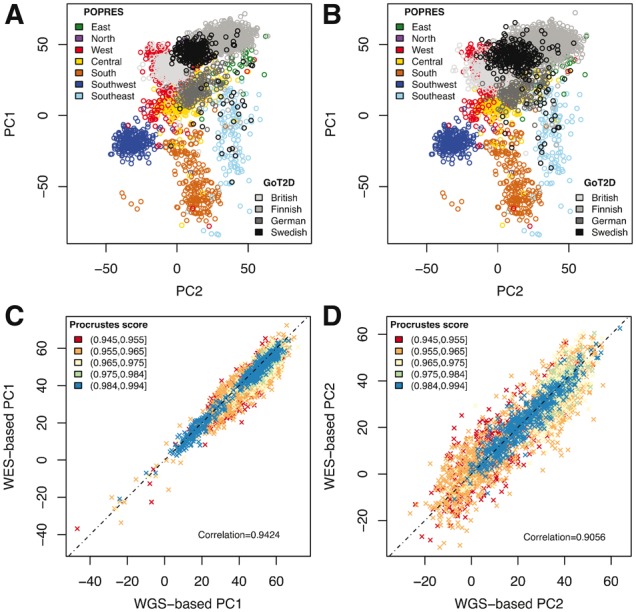
Ancestry estimation on the POPRES European map for 2335 samples from the GoT2D study. (**A**) Estimates using genotypes from 5X WGS data. (**B**) Estimates using sequence reads from 80X WES data. The overall Procrustes similarity score between (A) and (B) is *t_0 _*=_* *_0.9198. (**C**) Comparison of PC1 derived from WGS and WES data. (**D**) Comparison of PC2 derived from WGS and WES data. Points in (C) and (D) are colored based on the individual-specific Procrustes score *t* in the WES analysis

Among the 12 940 individuals, we also have whole genome sequence data (WGS, ∼5X) for 2335 Europeans from the GoT2D study, including British, Finnish, German and Swedish. We placed these individuals on a European ancestry map based on the POPRES panel. The results based on genotypes from WGS data and sequence reads from WES data are highly similar (Procrustes similarity *t_0 _*=_* *_0.9198, Pearson correlation 0.9424 for PC1 and 0.9056 for PC2; Fig. 2), with GoT2D samples cluster nicely with populations from their geographic regions. This example demonstrates that LASER can provide comparable ancestry estimates based on different types of data. The WES-based results are noisier than the WGS-based results due to the small number of targeted SNPs and low coverage across off-target regions in the WES data; the concordance between WES- and WGS-based results increases for samples with higher individual-specific Procrustes score *t* (Fig. 2). In practice, users can filter samples with insufficient data for ancestry estimation based on *t*. We note that by using a reference panel, LASER is more robust to the sampling distribution than standard PCA, for which uneven sampling of populations can distort top PCs (McVean, 2009). In our example, standard PCA cannot separate British, German and Swedish by PC1 and PC2 because Finnish has much larger sample size than the other populations and thus drives the first two PCs (Supplementary Fig. S2).

The LASER server parallelizes ancestry estimation and the total runtime for each job depends on the number of avaible CPUs. Ancestry estimation for a single study individual takes from a few seconds to several minutes, depending on the input data type (genotypes or sequence reads), the sample size of the ancestry reference panel, and the number of SNPs used in the analysis (Supplementary Table S2).

## 4 Conclusion

With a unified analysis framework and preprocessed ancestry reference panels, the LASER server allows users to map genotyped or sequenced samples from different studies into a common ancestry space without pooling the raw data. The ancestry estimates are directly comparable across studies, and thus can facilitate collaborations and help identify ancestry-matched external controls to boost power in disease studies.

## Funding

This research was supported by National Institutes of Health (NIH) grants HG000376 (to MB), HG007022 (to GRA) and by the Agency for Science, Technology and Research (A*STAR) in Singapore (to CW).

*Conflict of Interest*: none declared.

## Supplementary Material

Supplementary Data
