# Supplementary material for: LASER server: ancestry tracing with genotypes or sequence reads
Source: Bioinformatics. 2017 Feb 14;33(13):2056–8. doi: 10.1093/bioinformatics/btx075 (PMC5870850; doi:10.1093/bioinformatics/btx075)
Supplement: Supplementary Data [file btx075_supp.pdf]

LASER server: ancestry tracing with genotypes or sequence reads

Supplementary Data

The LASER method

For each ancestry reference panel of N individuals, LASER applies principal components analysis (PCA) on autosomal SNPs to construct a K-dimensional reference ancestry space. This K-dimensional space defines a common ancestry coordinate system for samples from different studies. Users can specify the value of K based on the reference panel and their research objectives. We typically choose K such that major ethnic groups or populations of interest are well separated. LASER allows genotypes or sequence reads for the study samples, and projects them into the reference ancestry space one by one.

To assign coordinates to a single genotyped individual, LASER uses SNPs shared between this individual and the N reference panel members to perform a PCA of the N+1 individuals and obtains the top K'≥K PCs. In general, larger values of K' lead to more accurate ancestry estimates because information from higher order PCs is used. However, when K' is too large (close to N), LASER may suffer from overfitting, leading to poor estimation accuracy. For example, when using the HGDP reference panel, we set the default values as K=4 because major continental groups are well separated in the top 4 PCs. Users can set K>4 for the HGDP reference panel if they are interested in intra-continental population structure such as separating different European populations. Alternatively, we recommend using a continental reference panel, such as the POPRES dataset for Europe, for easier interpretation of the results. We set K'=20 because we have found this provides good results similar to K'>20 and avoids the risk of overfitting (see simulation results in Wang *et al.* 2015). LASER then performs a projection Procrustes analysis (Gower and Dijksterhuis 2004) to find a set of transformations (projection, translation, rotation, reflection, and scaling) that project the N reference individuals from the K'-dimensional space to a K-dimensional space. The transformations maximize the Procrustes similarity between the projected coordinates and the pre-defined ancestry coordinates for reference samples. Finally, LASER uses these transformations to place each study individual into the K-dimensional reference ancestry space. The accuracy of the placement is partly reflected by the Procrustes similarity, which we denote as the individual-specific Procrustes score *t*.

When analyzing a sequenced individual, LASER simulates read counts for each reference individual conditional on its observed genotypes. The simulated data matches the sequencing depth and estimated per base error rate of the individual being placed (Wang *et al.* 2015). The simulated read data for reference individuals and observed read data of the study individual are then combined to obtain the top K' PCs of the N+1 individuals. Using these PCs, the analysis proceeds as with genotype data.

As long as the same reference panel is used, LASER maps all study individuals to the same K-dimensional ancestry space, regardless of differences in the available data types and variant sets.

Evaluating appropriateness of an ancestry reference panel

When an individual's ancestry is not represented in the reference panel, LASER might cluster the individual with reference populations of distant genetic background, yielding misleading results (Wang *et al.* 2015). To illustrate this point, we randomly selected 1000 individuals from the POPRES dataset as a European reference panel and use our LASER method to place the remaining 385 POPRES individuals (based on 306,469 genotyped SNPs) and all the HGDP individuals (based on 79,583 overlapping SNPs) on the European map (K'=20, K=2). Results are shown in **Figure S1A-C**. Both HGDP Europeans and POPRES test individuals were clustered with their geographic neighboring populations on the POPRES reference map, however, the placement of HGDP non-Europeans was misleading (**Figure S1B**). For example, HGDP individuals from Oceania were clustered with POPRES Italians, and HGDP East Asians overlapped with Southeastern Europeans in the POPRES reference panel. In this section, we propose a new statistic Z to capture such artifacts caused by using an inappropriate reference panel that doesn't represent ancestry background of the study individual.

Recall that LASER analyzes each study individual independently together with a set of N reference individuals using PCA followed by projection Procrustes analysis. The PCA is performed by eigen value decomposition on a (N+1)×(N+1) genetic relationship matrix $\boldsymbol{M}$, where each of the diagonal elements is the variance of the normalized genotypic values (or the normalized reference allele read counts for analyzing sequence reads) of an individual, sum across all loci. Details of the calculation of $\boldsymbol{M}$ can be found in our previous papers (Wang *et al.* 2014, 2015). We denote the last diagonal element of $\boldsymbol{M}$ as m_s, which is the variance for the study individual, and the first N diagonal elements as m_i $(i = 1,2, \dots, N)$, which are the variance for the N reference individuals. If the ancestry of a study individual is represented in the reference panel, m_s should have similar values to its neighboring reference individuals. We therefore propose the following approach to calculate a statistic indicating if the ancestry reference panel is appropriate for a study individual.

1. Identify k nearest reference individuals of a study individual based on Euclidean distances in the reference ancestry space. We set k=10 as the default value.
2. Calculate the mean and standard deviation of m_i for the k nearest neighbors (i.e., $i \in \{\text{indices of } k \text{ nearest neighbors}\}$), denoted as μ_{kNN} and σ_{kNN}, respectively.
3. Calculate Z score as $Z = \frac{m_s - \mu_{kNN}}{\sigma_{kNN}}$.

If the study individual has similar ancestry background as his k nearest neighbors, we will expect Z score to be close to 0. We evaluated the proposed Z score in our previous illustrative experiment. As shown in Figure S1D, majority of the POPRES test individuals and HGDP Europeans have Z<4. In contrast, HGDP individuals from East Asia, Oceania, America, and Africa all have Z>11, suggesting the POPRES reference panel is inappropriate for these samples. HGDP individuals from Middle East and Central South Asia have mean Z scores of 9.5 and 7.9 respectively, reflecting their close genetic relationship to Europeans compared to other non-European populations. The mean and standard deviation of the Z scores for different regions are summarized in **Table S1**. Overall, our proposed Z score serves as a good measurement to reflect how well a study individual's ancestry is reflected in the ancestry reference panel. We recommend users to be cautious in interpreting LASER results when Z score is greater than 4 or appears to be an outlier among all study samples.

References

Gower, J.C. and Dijksterhuis, G.B. (2004) *Procrustes Problems*. Oxford University Press, Oxford, New York.

Wang, C. *et al.* (2014) Ancestry estimation and control for population stratification for sequence-based association studies. *Nat Genet* **46**: 409-415.

Wang, C. *et al.* (2015) Improved ancestry estimation for both genotyping and sequencing data using projection Procrustes analysis and genotype imputation. *Am J Hum Genet*, **96**: 926-937.

Supplementary Tables

Table S1. Summary of Z scores for estimating ancestry of individuals from different geographic regions in the POPRES European reference ancestry space.

Region/ dataset	Number of individuals	Z score mean (±sd)
POPRES	385	2.1 (±1.2)
Europe	156	2.7 (±1.7)
Middle East	160	9.5 (±3.5)
C/S Asia	200	7.9 (±5.4)
East Asia	229	31.2 (±11.1)
Oceania	28	37.6 (±8.8)
America	63	41.5 (±5.5)
Africa	102	57.1 (±10.8)

Table S2. Computational time required to complete ancestry inference analysis with the LASER server (excluding download and format check).

Dataset	Input data type	Ancestry reference panel	No. of overlapping SNPs	Computational time per individual
T2D-GENES/GoT2D 80X WES	Genotypes	HGDP	12,719	8 seconds
GoT2D 5X WGS	Genotypes	POPRES	294,217	37 seconds
GoT2D 80X WES	Sequence reads	Imputed POPRES	4,212,452	543 seconds

Supplementary Figures

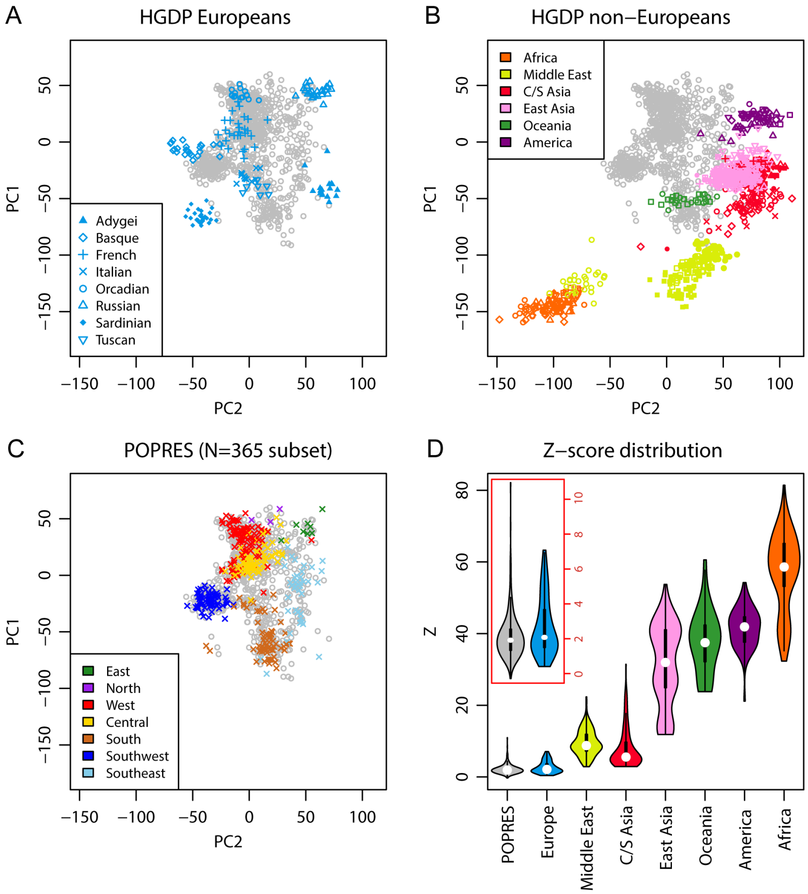

Figure S1. Ancestry estimation of HGDP individuals and a test set of 365 POPRES individuals using a European reference panel of 1,000 POPRES individuals. In panels A-C, colored points represent study individuals and grey points represent reference individuals. (A) Placement of HGDP Europeans. (B) Placement of HGDP non-Europeans. (C) Placement of POPRES test individuals. (D) Violin plot of Z scores for individuals from different regions. The red embedded box includes a zoom-in visualization of the Z scores for POPRES and HGDP Europeans.

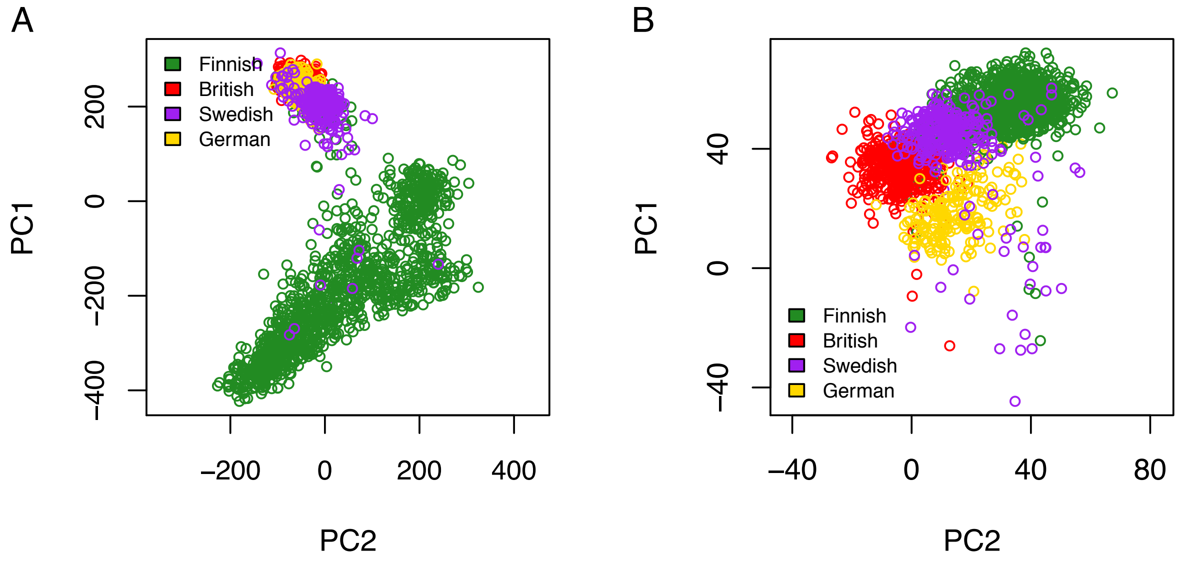

Figure S2. Comparison of standard PCA against LASER using whole genome sequence data for 2,335 Europeans (1,336 Finnish, 471 British, 341 Swedish, 187 German) from the GoT2D study. (A) Standard PCA: top two PCs were dominated by Finish population that had largest sample size. (B) LASER analysis using POPRES reference panel (reference individuals not shown). The Procrustes similarity t_0 score between PCA and LASER results was 0.52.
